# Is uterine artery embolisation a safe and effective modality to treat submucosal fibroids?

**DOI:** 10.1186/s42155-025-00546-x

**Published:** 2025-05-28

**Authors:** Rayhab Mashal, Neeral Patel, Bhavna Pitrola, Thomas Sewell, Asmaa Al-Kufaishi, Shabnam Taheri, Mohamad Hamady

**Affiliations:** 1 Department of Interventional Radiology, St Mary’s Hospital, Imperial College Healthcare NHS Trust, Praed Street, London, W2 1 NY UK; 2 Department of Obstetrics and Gynaecology, St Mary’s Hospital, Imperial College Healthcare NHS Trust, Praed Street, London, W2 1 NY UK

## Abstract

• Uterine fibroid embolization (UFE) reduced the median volume of dominant submucosal leiomyomas by 64% and achieved over 90% devascularization in 94.8% of cases.

• High patient satisfaction was observed, with 84.5% of patients successfully discharged without needing further intervention.

• Severe adverse events were rare, affecting only 3.2% of patients, with pelvic infections being the most significant.

• Mild adverse events occurred in 16.8% of patients, including infections requiring antibiotics and persistent vaginal discharge.

• A multidisciplinary approach is essential for managing patients with submucosal leiomyomas undergoing UFE to ensure optimal treatment outcomes.

## Introduction

Uterine leiomyomas are classified according to their location into three subtypes: submucosal, intramural, and subserosal leiomyomas [1, 2]. Submucosal leiomyomas are further classified into Type 0, which are mostly within the endometrial cavity; Type 1, which extend into the endometrial cavity with less than 50% of their components being intramural; and Type 2 leiomyomas, which have more than 50% of their components in the intramural region [2].

Among the different types of leiomyomas, submucosal leiomyomas are often regarded as the most troublesome variety because of their association with abnormal and often very heavy menstrual bleeding, along with reproductive issues such as infertility, recurrent miscarriages, or premature labour [3–5].

First introduced in 1976, hysteroscopic transcervical resection of submucosal leiomyoma is widely recognised the gold standard treatment for symptomatic submucosal leiomyomas and is considered a simple, safe, highly effective and minimally invasive (usually a day-case) procedure [6, 7]. However, there are limitations associated with this, including the size, number and location of the leiomyomas as well as the depth of invasion into the myometrium. If the submucosal leiomyomas are too large or deeply embedded within the myometrium, an abdominal myomectomy is usually considered [6, 7]. Laparoscopic myomectomy is associated with a faster recovery and is preferred, if feasible, but less ideal for patients with a greater number or larger fibroids [8]. However, as with TCRF, there are also limitations with abdominal myomectomy including an increased risk of intra-uterine adhesion formation which may affect fertility and/or an increased risk of perinatal complications [6, 7]. Data suggests that the risk of intra-uterine adhesion formation was as high as 31% following resection of a single leiomyoma and up to 45% for multiple leiomyomas [9].

Although a substantial body of evidence exists supporting UFE as an effective treatment option for patients with symptomatic leiomyomas, the role of UFE in patients with submucosal leiomyomas remains controversial, especially among the gynecologial community [10–14]. The main concerns are increased risk of pelvic infection, expulsion or prolapse of the leiomyoma into the endometrial cavity and persistent vaginal discharge [4, 6, 7]. There are limited studies that have evaluated the efficacy and outcome of UFE on submucosal leiomyomas specifically. The aim of this study was to evaluate the contemporary outcomes of UFE in patients with submucosal leiomyomas treated at a tertiary referral centre.

## Materials & methods

The local Ethics Committee waived the need for approval for the registration and use of retrospective analysis of anonymized data. Each patient signed an informed consent form for the intervention.

Information was collected from the local Radiology Information System (RIS), the Picture Archiving and Communication System (PACS), and the comprehensive, integrated electronic health record system (Cerner Millennium®, Oracle Health).

### Patient demographics

Data were collected from 155 patients identified with submucosal leiomyomas who underwent UFE between January 2013 and December 2022. The average age of the patients was 47.3 years (range 34–59 years).

### Uterine and fibroid data collection and follow-up

All patients underwent preoperative MRI examinations of the pelvis. We utilised T2-weighted sequences (sagittal and axial), which are part of our MRI gynaecological fibroid protocol, to obtain various measurements. The collected data included size (craniocaudal (CC) x anteroposterior (AP) x transverse (TS) dimensions) and volume (CC x AP x TS × 0.52) of the uterus and the dominant submucosal fibroid both pre- and post-embolization. The post-IV contrast T1-weighted images in the sagittal plane were used to estimate the percentage of devascularization. The study recorded the number of submucosal, intramural, and subserosal leiomyomas for each patient. It then focused primarily on the volume and size of the submucosal leiomyoma.All patients had a follow-up post contrast Magnetic Resonance Imaging (MRI) study to review devascularization of leiomyomas, approximately 6 months post-UFE. They were also reviewed in the Gynaecology and Interventional Radiology clinics.

### Criteria for technical and clinical success

The procedure was technically standardised. Super selective catheterisation of the uterine arteries was performed using a coaxial microcatheter technique. The embolic particles used were Contour™^P^ polyvinyl alcohol (Boston Scientific), with particle sizes of 355–500 microns or 500–710 microns, or Embozene™ microspheres (Boston Scientific), with particle sizes of 500–700 microns or 700- 900 microns.

The procedure was considered technically successful if blood flow was significantly reduced in both uterine arteries to 10 heartbeats. If only one uterine artery was successfully embolised, it is deemed a technical failure, except in cases where only a single uterine artery existed [15]. Clinical success was also recorded. Clinical success was defined as the resolution or satisfactory improvement of the patients presenting symptoms, such as menorrhagia or bulk-related pain, bloating, urinary urge, or constipation, without additional therapy [15].

### Procedural adverse outcomes and further interventions

Post-procedural adverse outcomes were categorized into two primary groups. The first group comprised patients who experienced adverse events, which were documented and classified as mild, moderate, or severe based on the Society of Interventional Radiology (SIR) adverse event classification system [16]. Mild adverse events included persistent vaginal discharge beyond 8 weeks [17] or a pelvic infection requiring antibiotics alone. Severe adverse events encompassed pelvic infections that necessitated either a TCRF or an emergency total abdominal hysterectomy.

The second group comprised patients who required additional procedures. This group included those needing a second UFE procedure due to only one uterine artery being embolized during the initial treatment, as well as patients needing either a planned or unplanned TCRF, a myomectomy, or a hysterectomy for persistent symptoms. A planned TCRF was considered during the initial consultation when a large submucosal leiomyoma was identified, and both the consulting Interventional Radiologist and Gynecologist deemed UAE beneficial prior to the TCRF. A large fibroid was subjectively determined to be ≥ 10 cm.

Detailed information was gathered, including the timing, type of reintervention, clinical outcome, and length of hospital stay.

### Statistical analysis

Discrete variables were presented as patient counts and percentages, while continuous variables were expressed as mean ± standard deviation. Differences between groups were evaluated using the paired t-test. Data collection was conducted with Excel (Microsoft, Washington, US), and statistical analysis was performed using GraphPad Prism (GraphPad LLC, San Diego, US). A *P-*value of less than 0.05 was considered statistically significant.

## Results

### Patient symptoms and prior procedures

From January 2013 to December 2022, a total of 724 patients underwent UFE for symptomatic uterine leiomyomas. Among these, 155 patients were identified as having submucosal leiomyomas.

Symptoms included heavy menstrual bleeding with clots in 134 patients (86%), prolonged menstrual cycles in 14 patients (9%), irregular menstrual cycles in 46 patients (30%), urinary frequency and urgency in 102 patients (66%), vaginal discharge in 50 patients (3.2%), exhaustion in 118 patients (76%), bloating in 132 patients (85%), and constipation in 15 patients (9.6%).

Nineteen (12%) of the patients had previously undergone myomectomy, 10 (6.5%) had a prior TCRF, and 4 (2.6%) had undergone hysteroscopy in the past.

### Uterine and fibroid data

**Fig. 1:**
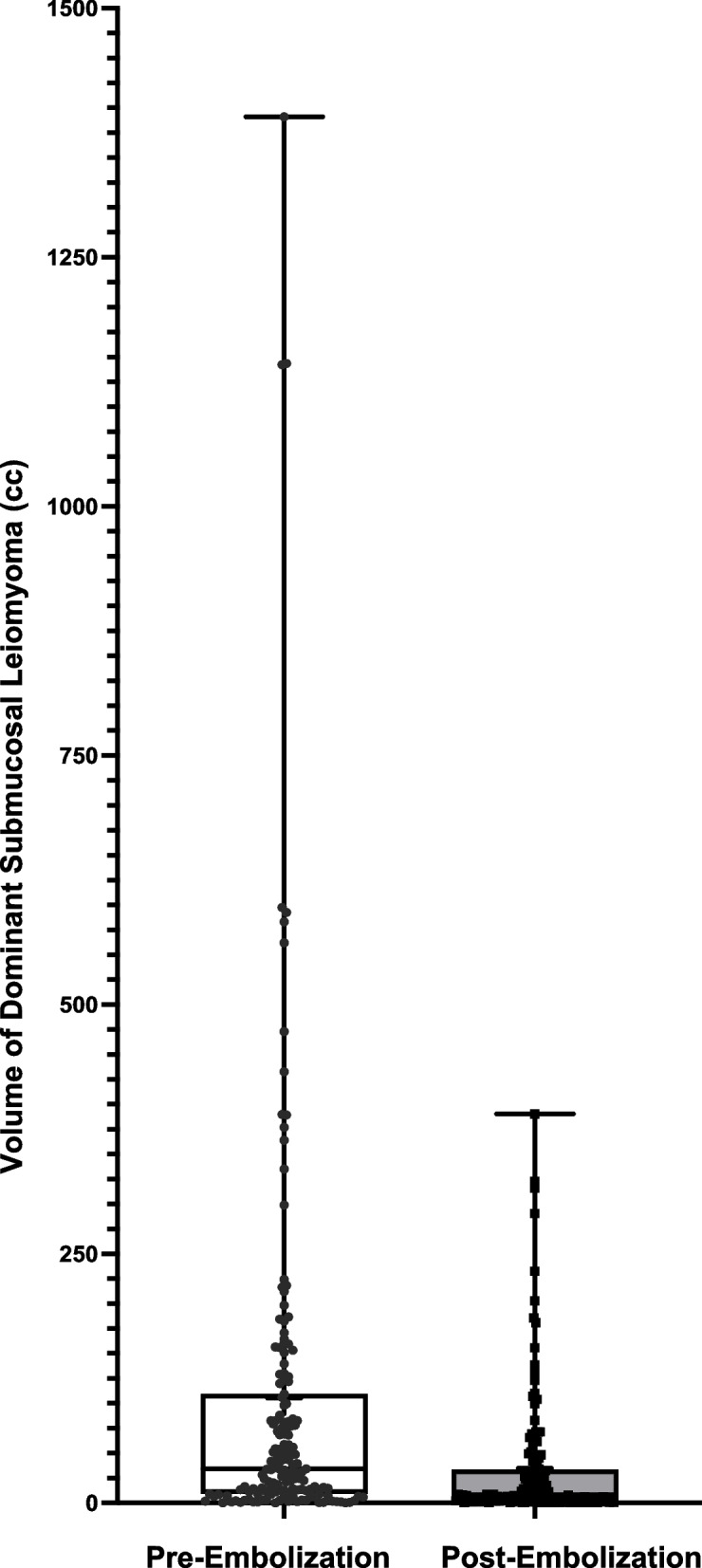
Volume of dominant submucosal leiomyoma pre- and post-embolization

**Fig. 2:**
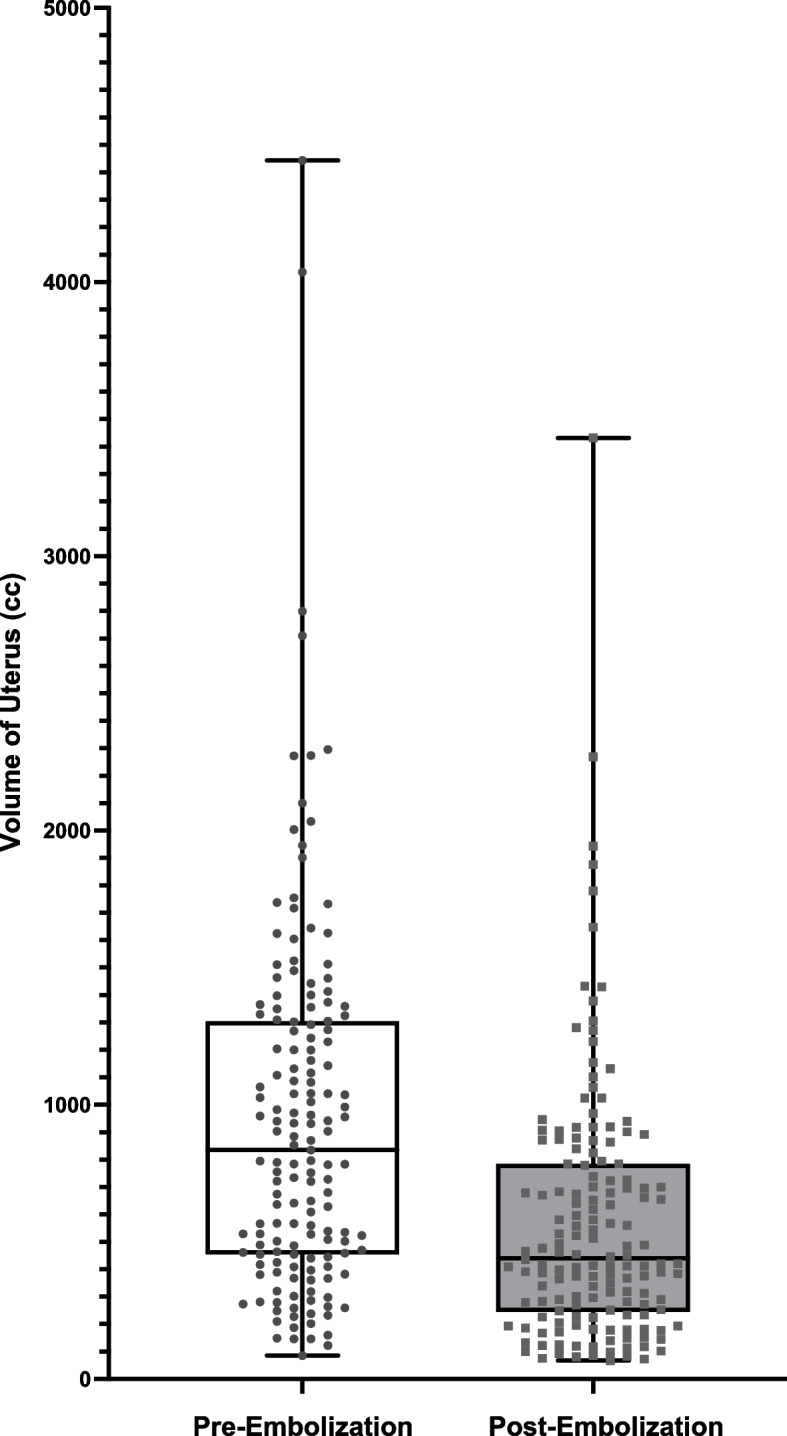
Volume of uterus pre- and post-embolization

Table 1, Figs. 1 and 2 compare the volumes of uterine and dominant submucosal leiomyomas before and after embolization. The median uterine volume pre-embolization was 834.9 cc (range 85.0–4444.0 cc), with a post-embolization median volume of 440.4 cc (range 67.1–3432.0 cc). The median percentage decrease in uterine volume was 39.9% (range 0.2–91.0%).

**Table 1:** Comparison of Uterus Volume and Dominant Submucosal Leiomyoma Volume Pre- and Post-Embolization. Review of Successful Devascularization Post-Embolization of Different Leiomyomas. Review of the Number of Submucosal Leiomyomas in Each Patient

		**Pre-Embolization**	**Post-Embolization**	**Change (%)**	***P-*Value**
**Uterus Volume (cc)**	Median	834.9	440.4	39.9	< 0.0001
	Median CI 95%	680.2–970.6	388.2–543.6	36.1–43.3	
	Range	85.0–4444.0	67.1–3432.0	0.2–91.0	
	Mean	949.2 ± 54.2	574.3 ± 38.1	39.0 ± 1.6	
	Mean CI 95%	842.0–1056.3	499.0–649.6	36.0–42.0	
**Dominant Submucosal Volume (cc)**	Median	34.20	8.3	64.0	< 0.0001
	Median CI 95%	24.2–51.3	5.7–13.9	57.7–69.7	
	Range	0.1–1391.0	0.01–390.4	1.5–99.7	
	Mean	105.5 ± 16.2	34.9 ± 5.4	60.9 ± 2.2	
	Mean CI 95%	73.6–137.4	24.3–45.5	56.5–65.3	
**Number of Submucosal Leiomyomas**	Median	2.0			
	Median CI 95%	2.0–3.0			
	Range	1.0–10.0			
	Mean	2.7 ± 0.2			
	Mean CI 95%	2.4–3.0			
**Devascularization of Submucosal Fibroids (%)**	Median		100		
	Median CI 95%		100—100		
	Range		0–100		
	Mean		96.09 ± 1.5		
	Mean CI 95%		93.2–99.0		
**Devascularization of Other Fibroid Subtypes – Intramural/Subserosal (%)**	Median		100		
	Median CI 95%		100—100		
	Range				
	Mean		95.25 ± 1.3		
	Mean CI 95%		92.8–97.8

Patients presented with 1 to 10 submucosal leiomyomas (median 2). For intramural leiomyomas, the number ranged from 0 to 50 (median 10). Subserosal leiomyomas varied between 0 and 13 (median 2). The median pre-embolization volume of the dominant submucosal leiomyoma was 34.20 cc (range 0.1–1391.0 cc), while the post-embolization volume was 8.3 cc (range 0.01–390.4 cc). The median percentage decrease in volume was 64% (range 1.5—99.7%).

### Technical success

Effective devascularization of submucosal fibroids exceeding 90% was achieved in 94.8% of patients. Likewise, 94.3% of patients obtained over 90% devascularization of the subserosal/intramural leiomyomas.

### Complications

Table 2 outlines the observed complications. Twenty-one patients (13.5%) reported persistent vaginal discharge after UFE. While vaginal discharge following UFE is common, it typically resolves within 8 weeks [17]. Therefore, if a patient continued to experience this after two months, it was recorded as a complication.

**Table 2:** Review of Complications and Further Procedures

	**Number of Patients**	**Percentage (%)**	**Interval between UAE and Second Procedure (Days) Median [Range]**
**Complications – Requiring Emergency Treatment**			
**Mild Adverse Event (A) (38)**			
Vaginal Discharge	21	13.5	-
Pelvic Infection –Antibiotics only	5	3.2	11 [11–14]
**Severe Adverse Event (C) (38)**			
Pelvic Infection –TCRF	3	1.9	56 [5–81]
Pelvic Infection –Total Abdominal Hysterectomy	2	1.3	6, 26
**Further Procedures – Persistent Symptoms Post-UAE**			
Second UAE	2	1.3	305, 720
Planned TCRF Post-UAE	3	1.9	62 [4–417]
Unplanned TCRF	5	3.2	369 [97–538]
Myomectomy	1	0.6	563
Hysterectomy	8	5.1	1188 [340–1455]
Symptoms Improved – Discharged	131	84.5	-

One patient started hormonal treatment (Desogestrel) as her symptoms (menorrhagia) were persistent post-UFE. Her symptoms have improved and she has since been discharged from clinic

One patient had persistent symptoms post-UFE, but decided against any further intervention or medical management

Overall, 10 patients (6.5%) developed pelvic infections (Table 3). Five patients (3.2%) were successfully treated with antibiotics alone, three patients (1.9%) required a TCRF, while two patients (1.3%) underwent an emergency total abdominal hysterectomy (TAH) for an infected, necrotic fibroid. One of the patients who required an emergency TCRF did not respond to two courses of antibiotic treatment. She was initially treated with intravenous antibiotics 60 days post-UFE. The patient returned to the Emergency Department 80 days post-UFE with offensive vaginal discharge and urinary retention and was found to have a protruding necrotic fibroid. The patient underwent an eventful TCRF the following morning.

**Table 3:** Details of patients with pelvic infection

Patient number	Presenting Complaint	Days after UAE	Microbiology Results			Antibiotic, Route (per oral PO, intravenous IV) and Duration	Admission into hospital and how many days in hospital	Outcome	Outcome Fibroid	Embolic size (Red = Right, Blue = Left)	Embolic amount(vials)
			High Vaginal Swab	Urine Microbiology Culture and Sensitivity (MC + S)	Blood Culture						
Pelvic Infection – Requiring Antibiotics Only											
Patient 1	Right lower abdominal pain, fever and dark brown PV discharge	2	Candida Albicans	No growth	No growth	IV Cefuroxime and Metronidazole 2/7 Stepped down to PO Co-amoxiclav for 7 days	Yes – 2	Discharged on oral antibiotic Co-amoxiclav 7-days course	Discharged from clinic	PVA 355–500	2.5
										PVA 500–710	2
										PVA 355–500	2
Patient 1	Fever, vomiting and minimal PV bleeding	13	Yeasts isolated	No growth	No growth	IV Cefuroxime and Metronidazole 3/7	Yes—3	Discharged on oral Cefalexin and Metronidazole 7-day course	Discharged from clinic	PVA 355–500	No record
Patient 2	Fever	9	No growth	No growth	No growth	-	No	-	Discharged from clinic	PVA 355–500	12
Patient 3	Sepsis post-procedure Fever	4	No growth	No growth	No growth	IV Cefuroxime and Metronidazole PO Ciprofloxacin	14	Cefalexin, Ciprofloxacin and Metronidazole – 7-day course	Discharged	PVA 355–500	6
										PVA 500–710	1
Patient 4	Fever, prolapsing fibroid *** Seen in ED and GP 4 times in five months with four courses of antibiotics*** (presented to two different hospitals at 28 days then 112 days post procedure)	28	Staphylococcus aureus	No growth	No growth	IV antibiotics on first presentation at different hospital	IV Co-amoxiclav	Fibroid passed PV Discharged on PO Co-amoxiclav 2/52	Discharged from clinic	PVA 355–500	3
										PVA 355–500	3
										PVA 500–710	1
Patient 5	Fever	11	-	-	-	IV Cefuroxime and Metronidazole	Yes—5	Discharged on PO antibiotics 2/52	Discharged from clinic	355–500	4
										500–710	1
Pelvic Infection – Requiring TCRF											
Patient 6	Fever and abdominal pain	Attended different hospital ED with avulsed fibroid Re-attended ED for TCRF	No record	No record	-	-	-	-	Discharged from clinic	700 Embozene Microspheres	2
										700 Embozene Microspheres	2
Patient 7	Fevers and abdominal pain	60	No growth	No growth	No growth	IV Ceftriaxone and Metronidazole + STAT Amikacin 3/7	Yes—3	Discharged on oral Clindamycin & Ciprofloxacin for 14 days	TCRF for necrotic fibroid-followed by discharge from clinic	355–500	1 vial
										500–710	1 vials
	Fevers and large necrotic fibroid prolapsing through cervix	80	Anaerobes + + + isolated	No growth	No growth	IV Co-amoxiclav 2/7	Yes—2	Discharged on oral Metronidazole and Ciprofloxacin for 7 days	Discharged from clinic	PVA 355–500	1vial
										PVA 500–710	1vial
Patient 8	Fever	20	No record	No record	No record	No record	No record	-	Discharged from clinic	Embozene Microspheres 700	2
										Embozene Microspheres 700	2
Pelvic Infection – Requiring Emergency TAH & BSO											
Patient 9	Fever, abdominal pain	26	Scanty anaerobes	Not performed	Not performed	IV Cefuroxime and Metronidazole escalated to IV Meropenem	Yes – 14 days	TAH	TAH Histopathology: Histopathology: ischaemic and necrotic change, acute inflammation suggestion abscess	PVA 355–500	2 vials
										PVA 355–500	2 vials
Patient 10	Fever, abdominal pain, prolapsing fibroid (21 cm)	6	Gram negative bacilli—Escherichia coli	Gram negative bacilli—Escherichia coli	Gram negative bacilli—Escherichia coli	IV Ceftriaxone and Metronidazole and Gentamicin	Yes – 12 days	TAH Discharged on oral Co-amoxiclav 7 days	TAH Histopathology: Uterus shows changes following UAE and secondary acute inflammation. Single leiomyoma—active endometritis following UAE. The leiomyoma has prolapsed through the cervix	PVA 355–500	3 vials
										PVA 355–500	3 vials

Four patients were noted to pass their dominant submucosal leiomyoma per vagina, without any further intervention.

Table 2 illustrates the interval between the initial UFE procedure and the onset of complications. Patients treated with antibiotics alone for pelvic infections presented between 1 and 14 days post-UFE. Three patients with pelvic infections underwent TCRF at 5, 56, and 81 days post-UFE, respectively. The two patients who required emergency TAH had their surgeries at 6 and 26 days post-UFE.

Three patients had a planned TCRF prior to UFE because of the large size (≥ 10 cm) of their submucosal leiomyomas. These patients underwent uncomplicated TCRF procedures between 4 and 417 days after UFE, with a median interval of 62 days.

Twenty patients reported persistent symptoms during their follow-up appointments in the gynaecology clinic. Two patients (1.3%) underwent a second UFE procedure at 305 and 720 days post-UFE. In both cases, only one uterine artery was embolized during the initial procedure due to vasospasm and failure to cannulate the contralateral artery.

Three patients chose to undergo TCRF due to persistent heavy menstrual bleeding, while two opted for the procedure because of ongoing troubling vaginal discharge beyond 8 weeks. These procedures were performed between 97 and 538 days post-UAE (Table 2). One patient’s TCRF was delayed due to cardiac issues. When the procedure was eventually performed, no intracavity or submucosal fibroid was found, and thus no resection was required.

One patient opted for a myomectomy, which was performed 563 days post-UFE. Eight patients opted for a total abdominal hysterectomy (TAH) due to persistent symptoms, with these procedures occurring between 340 and 1455 days post-UFE.

Two patients had further clinic reviews for ongoing symptoms. One patient with persistent menorrhagia chose conservative management with Desogestrel, which has effectively controlled her symptoms, leading to her discharge from the clinic. Another patient, who did not wish to pursue further treatment for her symptomatic leiomyomas, was also discharged from the clinic.

Overall, 131 patients were satisfied with their results after undergoing UFE and were discharged from the clinic without the need for any further intervention, including a second embolization. The remaining patients were reviewed in the clinic following their secondary interventions. All but two of these patients have since been discharged. The two patients still under clinic review are awaiting TAH due to persistent fibroid-related symptoms and need to lower their body mass index before being considered for the procedure.

## Discussion

A literature search was conducted to review studies evaluating complications in patients with submucosal leiomyomas following UFE. This search was challenging due to the limited amount of literature specifically addressing this subject, as most studies focus on all subtypes of leiomyomas. However, the most pertinent studies were reviewed and compared against our objectives and data. Table 4 summarizes the most relevant studies, highlighting their findings, conclusions, and limitations.

**Table 4:** Summary of relevant studies, highlighting the findings, conclusions and limitations

Study	Aim	Main Findings	Conclusion	Limitations
Boris-Radeleff et al. [Boris-Radeleff 2009] [18]	Retrospective review of 20 patients. Evaluated the frequency, probability and factors associated with expulsion of submucosal leiomyoma after UFE Reviewed technical and clinical results at their one-year follow up	Technical success in all 20 patients with 2 (10%) minor and 1 (5%) major complications Complications: ▪ Transient, post-procedural hypotension ▪ Small post-interventional inguinal haematoma ▪ One-day hospitalization for a patient with vaginal bleeding related to the expulsion of a dominant submucosal leiomyoma 10 (out of 20) patients expelled their dominant submucosal fibroid in a time frame of 4 weeks- 3 months post-UFE, 19 patients without complications	Statistically significant reduction in submucosal leiomyoma size and uterine volume All patients reported a reduction in their symptoms and were satisfied with their post-UFE results	A limited number of patients
Namkung et. al [Namkung 2018] [4]	Retrospective review of 8 patients Evaluating the safety and effectiveness of hysteroscopic myomectomy after UFE for large sized submucosal leiomyomas with deep myometrial invasion	Mean volume of the submucosal leiomyomas was 87.7 cm^3^, reviewed on MRI Reduction in volume after UFE was 83.3% UFE was followed by a hysteroscopic myomectomy and all women improved symptoms with no recurrence one-year later	Concluded that there were no complications observed in patients post-UFE	Study was not reviewing patients with submucous leiomyomas who exclusively had a UFE but patients who underwent hysteroscopic myomectomy following UAE
Al-Fozan et. al [Al Fozan 2002] [19]	Presented a number of cases of septic uterus leading to early hysterectomy	Five out of seven patients had submucous leiomyomas ▪ Three patients were found to have septic uterine necrosis at surgery ▪ Two patients had necrotic myomas	Al Fozan argued that despite some authors viewing myoma expulsion as a complication, it may be a natural phenomenon with submucous leiomyomas and a cervical culture pre-UAE is recommended to avoid performing UAE in the presence of infection	Small number of patients reviewed
Pelage et. al [Pelage 2000] [20]	Prospectively cohort of 6 patients with submucosal fibroids out of 80 patients, evaluated the effectiveness and safety of UFE in the management of symptomatic uterine leiomyoma	▪ Four women complained of necrotic fragments of a pedunculated submucosal leiomyoma expelled through the cervix during the first month post-procedure ▪ Only 1 patient (17%) underwent a hysterectomy due to acute septic uterine necrosis 17-days post-UAE. Although this was put down to a large submucosal leiomyoma, a size was not specified in this case Five patients reported no improvement of symptoms ▪ Four patients underwent further myomectomy ▪ One patient underwent a hysterectomy	Superselective arterial embolization of the uterine arteries is an effective means of controlling symptomatic uterine leiomyoma	Small cohort (although only one patient developed septic necrotic uterus) Results did not specify which patients had submucous leiomyomas Unclear which subtype of leiomyomas the five patients who did not experience any improvement had
Rajan et. al [Rajan 2004] [21]	Retrospective review of UFE performed over a three-year period to identify risk factors (including location and size of dominant leiomyoma) for the development of intrauterine infection following UFE	414 UFE procedures were performed in 410 patients 148 patients (36.1%) had submucosal leiomyomas 5 patients (3.4%) developed infectious complications and all were within the submucosal patient group ▪ 4 patients required antibiotics alone ▪ 5 th patient underwent a TCRF, however, during attempted hysteroscopic removal of the leiomyoma, the uterus was perforated and the patient underwent a hysterectomy	Significant difference in the occurrence of intrauterine infectious complications in patients with submucosal leiomyomas versus those without (*P* = 0.006) with univariate analysis Authors described this association was not significant with multivariate analysis, including embolic agent used (*P* = 0.71), quantity of embolic agent (*P* = 0.33), size (*P* = 0.74) and location (*P* = 1.0) of dominant leiomyoma and use of prophylactic pre-procedure antibiotics (*P* = 0.81) Authors concluded that of the variables examined, none were found to be predictive of developing intrauterine infectious complications	Using transvaginal ultrasound imaging to review size and location of the leiomyomas

The two main sources of evidence are Boris-Radeleff et al. [18] and Rajan et al. [21]. Boris-Radeleff et al. assessed the frequency, probability, and factors associated with the expulsion of submucosal leiomyoma following UFE, along with the technical and clinical outcomes at one-year follow-up. Technical success was achieved in all 20 patients, with 2 (10%) minor complications and 1 (5%) major complication. These complications included transient post-procedural hypotension, a small post-interventional inguinal hematoma, and a one-day hospitalization for a patient with vaginal bleeding due to the expulsion of a dominant submucosal leiomyoma. Although half of the patients experienced leiomyoma expulsion, this occurred without requiring medical intervention in 95% of cases (19 out of 20 patients). The study also revealed a statistically significant (*P* = 0.05) decrease in both the size of submucosal leiomyoma and overall uterine volume. All patients reported an improvement in their symptoms and expressed satisfaction with their post-UFE outcomes.

Rajan et al. [21] conducted a retrospective review of UFE procedures performed over three years to identify risk factors for intrauterine infection post-UFE. They assessed various factors, including the location and size of the dominant leiomyoma. The study involved 414 UFE procedures in 410 patients, with 148 patients (36.1%) having submucosal leiomyomas. Among these, 5 patients (3.4%) developed infectious complications, all of whom had submucosal leiomyomas. Four patients were treated with antibiotics alone, while the fifth required a TCRF. During the hysteroscopic removal of the leiomyoma, the uterus was perforated, necessitating a hysterectomy.

The incidence of intrauterine infectious complications was significantly higher in patients with submucosal leiomyomas compared to those without (*P* = 0.006), according to univariate analysis. However, multivariate analysis showed that this association was not significant when considering the embolic agent used (*P* = 0.71), the quantity of the embolic agent (*P* = 0.33), the size (*P* = 0.74) and location (*P* = 1.0) of the dominant leiomyoma, and the use of prophylactic pre-procedure antibiotics (*P* = 0.81). The authors concluded that none of the variables examined were predictive of developing intrauterine infectious complications. They noted limitations in their study, such as the relatively small sample size (414 patients) and the use of transvaginal ultrasound imaging to assess the size and location of the leiomyomas.

The literature addressing our primary objective is relatively sparse. Many of the studies referenced earlier either advise against performing UFE in patients with submucosal leiomyomas, present findings based on a small sample size, or fail to specifically distinguish between patients with submucosal leiomyomas and those with other types of fibroids. Consequently, there is a lack of comprehensive and detailed research that directly explores the outcomes and efficacy of UFE in the context of submucosal leiomyomas.

This study involved a larger sample size compared to those previously mentioned, encompassing 155 patients diagnosed specifically with submucosal leiomyomas out of a total of 724 patients who underwent UFE during the study period. Each patient’s leiomyomas were classified according to the FIGO classification system to ensure precise categorization. Both pre- and post-UFE evaluations were conducted using MRI, which offers superior accuracy compared to transvaginal ultrasound scanning [22].

This cohort demonstrated that UFE is a safe and effective treatment for patients with submucosal leiomyomas. The occurrence of pelvic infections was relatively low at 6.5%, with only five patients requiring additional surgical interventions such as TCRF or TAH.

The main limitation of the current study is the retrospective design. However, the patient cohort is drawn from the local catchment area, and all patient medical records are maintained in the hospital’s electronic Cerner system. Another limitation is that data collection focused on the volume of the dominant submucosal leiomyoma. Patients had between 1 and 10 submucosal leiomyomas, with a median of 2. Despite these limitations, the positive clinical outcomes observed in most patients suggest that the study has captured the most relevant findings.

In conclusion, this study demonstrates that UFE is a safe and effective treatment option for patients with symptomatic submucosal leiomyomas and is associated with high clinical success. Implementing clear pathways and adopting a multidisciplinary team approach is critical in managing these patients to ensure the best treatment options are presented.

## Data Availability

Available in the main manuscript.

## References

[1] Gomez E, Nguyen M, Fursevich D, Macura K, Gupta A. MRI-Based Pictorial Review of the FIGO Classification System for Uterine Fibroids. Abdom Radiol. 2021;46:2146–55. 10.1007/s00261-020-02882-z 33385249

[2] Goodwin SC, Walker WJ. Uterine artery embolization for the treatment of uterine fibroids. Curr Opin Obstet Gynecol. 1998;10:315–20. 9719883 10.1097/00001703-199808000-00006

[3] Buttram VC, Reiter RC. Uterine leiomyomata: etiology, symptomatology and management. Fertil Steril. 1981;36:4433–45. 10.1016/s0015-0282(16)45789-4 7026295

[4] Namkung J, Kang SY, Chung YJ, et al. Multidisciplinary Approach in Large-sized Submucosal Myoma: Hysteroscopic Myomectomy after Uterine Artery Embolization. J Minim Invasive Gynecol. 2019;26:643–7. 29969685 10.1016/j.jmig.2018.06.016

[5] Uimari O, Subramaniam KS, Vollenhoven B, Tapmeier TT. Uterine Fibroids (Leiomyomata) and Heavy Menstrual Bleeding. Front Reprod Health. 2022;4: 818243. 36303616 10.3389/frph.2022.818243 PMC9580818

[6] Hallez JP. Single-stage total hysteroscopic myomectomies: indications, techniques, and results. Fertil Steril. 1995;63:703–8. 7890051

[7] Vercellini P, et al. Hysteroscopic myomectomy: long-term effects on menstrual pattern and fertility. Obstet Gynecol. 1999;94:41–7. 10472856 10.1016/s0029-7844(99)00346-4

[8] Saridogan E. Surgical treatment of fibroids in heavy menstrual bleeding. Womens Health (Lond). 2016;12:53–62. 26693796 10.2217/whe.15.89 PMC5779570

[9] Taskin O, Sadik S, Onoglu A, et al. Role of endometrial suppression on the frequency of intrauterine adhesions after resectoscopic surgery. J Am Assoc Gynecol Laparosc. 2000;7:351–4. 10924629 10.1016/s1074-3804(05)60478-1

[10] de Bruijn AM, Ankum WM, Reekers JA, Birnie E, van der Kooij SM, Volkers NA, et al. Uterine artery embolization vs hysterectomy in the treatment of symptomatic uterine fibroids: 10-year outcomes from the randomized EMMY trial. Am J Obstet Gynecol. 2016;215:745. 10.1016/j.ajog.2016.06.051 27393268

[11] Edwards RD, Moss JG, Lumsden MA, Wu O, Murray LS, Twaddle S, et al. Uterine-artery embolization versus surgery for symptomatic uterine fibroids. N Engl J Med. 2007;356:360–70. 17251532 10.1056/NEJMoa062003

[12] Manyonda I, Belli A-M, Lumsden M-A, Moss J, McKinnon W, Middleton LJ, et al. Uterine-artery embolization or myomectomy for uterine fibroids. N Engl J Med. 2020;383:440–51. 32726530 10.1056/NEJMoa1914735

[13] Pinto I, Chimeno P, Romo A, et al. Uterine fibroids: uterine artery embolization versus abdominal hysterectomy for treatment–a prospective, randomized, and controlled clinical trial. Radiology. 2003;226:425–31. 12563136 10.1148/radiol.2262011716

[14] Toor SS, Jaberi A, Macdonald DB, et al. Complication rates and effectiveness of uterine artery embolization in the treatment of symptomatic leiomyomas: a systematic review and meta-analysis. AJR Am J Roentgenol. 2012;199:1153–63. 23096193 10.2214/AJR.11.8362

[15] Hovsepian DM, Siskin GP, Bonn J, et al. CIRSE Standards of Practice Committee; SIR Standards of Practice Committee. Quality improvement guidelines for uterine artery embolization for symptomatic leiomyomata. Cardiovasc Intervent Radiol. 2004;27:307–13. 15346204 10.1007/s00270-004-0087-4

[16] Baerlocher MO, Nikolic B, Sze DY. Adverse Event Classification: Clarification and Validation of the Society of Interventional Radiology Specialty-Specific System. J Vasc Interv Radiol. 2023;34:1–3. 36244632 10.1016/j.jvir.2022.10.011

[17] van Overhagen H, Reekers JA. Uterine artery embolization for symptomatic leiomyomata. Cardiovasc Intervent Radiol. 2015;38:536–42. 25465064 10.1007/s00270-014-1031-x

[18] Radeleff B, Eiers M, Bellemann N, Ramsauer S, Rimbach S, Kauczor HU, Richter GM. Expulsion of dominant submucosal fibroids after uterine artery embolization. Eur J Radiol. 2010;75:e57-63. 19692192 10.1016/j.ejrad.2009.07.013

[19] Al-Fozan H, Tulandi T. Factors affecting early surgical intervention after uterine artery embolization. Obstet Gynecol Surv. 2002;57:810–5. 12493983 10.1097/00006254-200212000-00005

[20] Pelage JP, Le Dref O, Soyer P, Kardache M, Dahan H, Abitbol M, Merland JJ, Ravina JH, Rymer R. Fibroid-related menorrhagia: treatment with superselective embolization of the uterine arteries and midterm follow-up. Radiology. 2000;215:428–31. 10796920 10.1148/radiology.215.2.r00ma11428

[21] Rajan DK, Beecroft JR, Clark TW, Asch MR, Simons ME, Kachura JR, Sved M, Sniderman KW. Risk of intrauterine infectious complications after uterine artery embolization. J Vasc Interv Radiol. 2004;15:1415–21. 15590799 10.1097/01.RVI.0000141337.52684.C4

[22] Medema AM, Zanolli NC, Cline B, Pabon-Ramos W, Martin JG. Comparing magnetic resonance imaging and ultrasound in the clinical evaluation of fibroids prior to uterine artery embolization. Curr Probl Diagn Radiol. 2024;53(2):308–12. 38267343 10.1067/j.cpradiol.2024.01.028

